# Supplementary material for: MEME-LaB: motif analysis in clusters
Source: Bioinformatics. 2013 May 14;29(13):1696–7. doi: 10.1093/bioinformatics/btt248 (PMC3694638; doi:10.1093/bioinformatics/btt248)
Supplement: Supplementary Data [file supp_btt248_Supplementary_Data.zip › Supplementary_Data/Results_Files/which_gene_ids/cluster39_motif1.html]

Cluster 39 Motif 1


Cluster 39 Motif 1 is found in the following promoters

| Gene ID | Strand | Position\* |
| --- | --- | --- |

|  |  |  |
| --- | --- | --- |
| AT1G01725 | - | 74/500 |
| AT1G03730 | + | 344/500 |
| AT1G05785 | + | 363/500 |
| AT1G06870 | + | 242/500 |
| AT1G08250 | + | 16/500 |
| AT1G08315 | + | 366/500 |
| AT1G08770 | - | 53/500 |
| AT1G09645 | + | 340/500 |
| AT1G10690 | + | 383/500 |
| AT1G12600 | + | 128/500 |
| AT1G12760 | + | 51/500 |
| AT1G12970 | + | 258/500 |
| AT1G13760 | - | 312/500 |
| AT1G14190 | + | 36/500 |
| AT1G14400 | + | 441/500 |
| AT1G14450 | - | 178/500 |
| AT1G15100 | + | 489/500 |
| AT1G15350 | - | 415/500 |
| AT1G15860 | + | 397/500 |
| AT1G16500 | + | 62/500 |
| AT1G18640 | - | 228/500 |
| AT1G20350 | + | 153/500 |
| AT1G21000 | + | 298/500 |
| AT1G21720 | - | 352/500 |
| AT1G23090 | - | 299/500 |
| AT1G23490 | + | 282/500 |
| AT1G24350 | + | 312/500 |
| AT1G26630 | - | 389/500 |
| AT1G27290 | + | 283/500 |
| AT1G27970 | + | 441/500 |
| AT1G29120 | + | 300/500 |
| AT1G29355 | - | 276/500 |
| AT1G29970 | + | 243/500 |
| AT1G32460 | - | 251/500 |
| AT1G32790 | + | 415/500 |
| AT1G33110 | + | 23/500 |
| AT1G36340 | + | 297/500 |
| AT1G44960 | + | 476/500 |
| AT1G47530 | - | 96/500 |
| AT1G47830 | + | 221/500 |
| AT1G48200 | + | 229/500 |
| AT1G48370 | + | 335/500 |
| AT1G50090 | + | 372/500 |
| AT1G50430 | - | 288/500 |
| AT1G51160 | - | 406/500 |
| AT1G53030 | - | 98/500 |
| AT1G54115 | + | 151/500 |
| AT1G55190 | + | 193/500 |
| AT1G55240 | + | 214/500 |
| AT1G55730 | - | 243/500 |
| AT1G57680 | - | 297/500 |
| AT1G61250 | - | 122/500 |
| AT1G61370 | - | 322/500 |
| AT1G62422 | + | 114/500 |
| AT1G66740 | - | 18/500 |
| AT1G68170 | - | 13/500 |
| AT1G68310 | - | 274/500 |
| AT1G68490 | - | 96/500 |
| AT1G71190 | + | 481/500 |
| AT1G72680 | - | 301/500 |
| AT1G72750 | - | 64/500 |
| AT1G73965 | - | 430/500 |
| AT1G74270 | - | 470/500 |
| AT1G74340 | - | 313/500 |
| AT1G75810 | + | 395/500 |
| AT1G76760 | - | 185/500 |
| AT1G76970 | - | 319/500 |
| AT1G78300 | + | 63/500 |
| AT1G78895 | + | 258/500 |
| AT1G79990 | + | 359/500 |
| AT2G01190 | + | 312/500 |
| AT2G04550 | + | 257/500 |
| AT2G05752 | - | 251/500 |
| AT2G07675 | - | 24/500 |
| AT2G07707 | - | 281/500 |
| AT2G07785 | + | 337/500 |
| AT2G15340 | - | 13/500 |
| AT2G15570 | - | 191/500 |
| AT2G19350 | - | 224/500 |
| AT2G19760 | - | 55/500 |
| AT2G21190 | - | 54/500 |
| AT2G22370 | + | 321/500 |
| AT2G24210 | - | 108/500 |
| AT2G24570 | + | 18/500 |
| AT2G25737 | + | 255/500 |
| AT2G25970 | + | 66/500 |
| AT2G27140 | - | 8/500 |
| AT2G27490 | - | 428/500 |
| AT2G28760 | - | 231/500 |
| AT2G29480 | - | 486/500 |
| AT2G31305 | - | 434/500 |
| AT2G33690 | + | 451/500 |
| AT2G35520 | + | 400/500 |
| AT2G35733 | + | 96/500 |
| AT2G36300 | + | 41/500 |
| AT2G36580 | + | 329/500 |
| AT2G37035 | + | 363/500 |
| AT2G37975 | + | 54/500 |
| AT2G39890 | - | 450/500 |
| AT2G40095 | + | 312/500 |
| AT2G41160 | + | 210/500 |
| AT2G41230 | - | 338/500 |
| AT2G41420 | + | 365/500 |
| AT2G42310 | + | 279/500 |
| AT2G44430 | + | 145/500 |
| AT2G44600 | - | 96/500 |
| AT2G46140 | + | 414/500 |
| AT2G46310 | + | 401/500 |
| AT3G02740 | - | 480/500 |
| AT3G03160 | - | 159/500 |
| AT3G03310 | + | 216/500 |
| AT3G03320 | - | 234/500 |
| AT3G03550 | + | 252/500 |
| AT3G05545 | + | 484/500 |
| AT3G05580 | - | 148/500 |
| AT3G06760 | + | 281/500 |
| AT3G07970 | + | 489/500 |
| AT3G08700 | - | 264/500 |
| AT3G08780 | - | 203/500 |
| AT3G10260 | - | 386/500 |
| AT3G10640 | - | 271/500 |
| AT3G11230 | + | 185/500 |
| AT3G11530 | + | 72/500 |
| AT3G12040 | + | 362/500 |
| AT3G12300 | - | 253/500 |
| AT3G12630 | - | 1/500 |
| AT3G12740 | + | 74/500 |
| AT3G13720 | - | 4/500 |
| AT3G13910 | + | 237/500 |
| AT3G14430 | - | 375/500 |
| AT3G14440 | - | 334/500 |
| AT3G16280 | + | 486/500 |
| AT3G16720 | + | 421/500 |
| AT3G19660 | + | 297/500 |
| AT3G20310 | + | 359/500 |
| AT3G20670 | + | 193/500 |
| AT3G20800 | - | 410/500 |
| AT3G21610 | + | 296/500 |
| AT3G21690 | + | 328/500 |
| AT3G21710 | - | 218/500 |
| AT3G22200 | - | 167/500 |
| AT3G22630 | - | 222/500 |
| AT3G22950 | - | 446/500 |
| AT3G23200 | + | 276/500 |
| AT3G23540 | + | 200/500 |
| AT3G24050 | - | 277/500 |
| AT3G25727 | - | 417/500 |
| AT3G29170 | + | 332/500 |
| AT3G47820 | - | 40/500 |
| AT3G52730 | - | 42/500 |
| AT3G52770 | - | 272/500 |
| AT3G52800 | - | 308/500 |
| AT3G53370 | + | 363/500 |
| AT3G54120 | + | 57/500 |
| AT3G54650 | + | 248/500 |
| AT3G54950 | + | 179/500 |
| AT3G55620 | - | 319/500 |
| AT3G56110 | + | 433/500 |
| AT3G57160 | + | 43/500 |
| AT3G57390 | + | 370/500 |
| AT3G57480 | + | 480/500 |
| AT3G58460 | + | 216/500 |
| AT3G59280 | + | 6/500 |
| AT3G60690 | + | 21/500 |
| AT3G61980 | - | 7/500 |
| AT4G00840 | + | 419/500 |
| AT4G01320 | - | 460/500 |
| AT4G03960 | + | 329/500 |
| AT4G05390 | + | 468/500 |
| AT4G05460 | - | 374/500 |
| AT4G10140 | - | 242/500 |
| AT4G10150 | - | 455/500 |
| AT4G10360 | + | 259/500 |
| AT4G12080 | - | 108/500 |
| AT4G12250 | - | 427/500 |
| AT4G12410 | - | 230/500 |
| AT4G14220 | - | 489/500 |
| AT4G14450 | - | 72/500 |
| AT4G15955 | + | 274/500 |
| AT4G16265 | - | 352/500 |
| AT4G16850 | - | 342/500 |
| AT4G17215 | + | 254/500 |
| AT4G17310 | + | 230/500 |
| AT4G17410 | + | 3/500 |
| AT4G19200 | - | 315/500 |
| AT4G19360 | - | 268/500 |
| AT4G19700 | - | 257/500 |
| AT4G19950 | + | 80/500 |
| AT4G20380 | + | 59/500 |
| AT4G20480 | + | 387/500 |
| AT4G21110 | - | 55/500 |
| AT4G21160 | - | 418/500 |
| AT4G21560 | + | 45/500 |
| AT4G23920 | + | 338/500 |
| AT4G27680 | + | 247/500 |
| AT4G27940 | - | 330/500 |
| AT4G27960 | + | 371/500 |
| AT4G28370 | + | 475/500 |
| AT4G28770 | + | 220/500 |
| AT4G32020 | + | 326/500 |
| AT4G32680 | - | 256/500 |
| AT4G33910 | + | 385/500 |
| AT4G35460 | - | 472/500 |
| AT4G35840 | + | 264/500 |
| AT4G39220 | - | 290/500 |
| AT4G39820 | + | 441/500 |
| AT5G01250 | + | 285/500 |
| AT5G01720 | + | 467/500 |
| AT5G03905 | - | 73/500 |
| AT5G04330 | - | 59/500 |
| AT5G04760 | - | 327/500 |
| AT5G05100 | + | 418/500 |
| AT5G05850 | - | 369/500 |
| AT5G06170 | + | 150/500 |
| AT5G06190 | + | 462/500 |
| AT5G06500 | + | 467/500 |
| AT5G08139 | - | 219/500 |
| AT5G08300 | - | 142/500 |
| AT5G09330 | - | 427/500 |
| AT5G09680 | - | 249/500 |
| AT5G09800 | - | 487/500 |
| AT5G11970 | + | 147/500 |
| AT5G15320 | + | 144/500 |
| AT5G17060 | + | 365/500 |
| AT5G17280 | + | 195/500 |
| AT5G17590 | + | 413/500 |
| AT5G20910 | - | 40/500 |
| AT5G22350 | - | 400/500 |
| AT5G23140 | + | 464/500 |
| AT5G25170 | + | 336/500 |
| AT5G25940 | + | 469/500 |
| AT5G26600 | + | 394/500 |
| AT5G27840 | - | 458/500 |
| AT5G27860 | + | 315/500 |
| AT5G33290 | + | 381/500 |
| AT5G33320 | + | 266/500 |
| AT5G35110 | + | 189/500 |
| AT5G37480 | - | 105/500 |
| AT5G39250 | - | 204/500 |
| AT5G40470 | - | 404/500 |
| AT5G41390 | + | 266/500 |
| AT5G43190 | - | 485/500 |
| AT5G44240 | - | 210/500 |
| AT5G45110 | - | 233/500 |
| AT5G46350 | + | 393/500 |
| AT5G47120 | - | 74/500 |
| AT5G48655 | - | 382/500 |
| AT5G51260 | + | 27/500 |
| AT5G51770 | - | 487/500 |
| AT5G52250 | + | 352/500 |
| AT5G53590 | + | 432/500 |
| AT5G53650 | + | 269/500 |
| AT5G54800 | - | 200/500 |
| AT5G55850 | + | 284/500 |
| AT5G56170 | + | 242/500 |
| AT5G56230 | - | 109/500 |
| AT5G57050 | - | 353/500 |
| AT5G58060 | - | 75/500 |
| AT5G58470 | + | 358/500 |
| AT5G60750 | - | 4/500 |
| AT5G64090 | - | 7/500 |
| AT5G64310 | + | 86/500 |
| AT5G65495 | + | 265/500 |
| AT5G67350 | + | 283/500 |
| AT5G67490 | + | 393/500 |
| AT5G67590 | - | 483/500 |

**\*** Overlapping sequences are merged to avoid redundancy. As a result some sequences in the final set may be longer than the specified maximum.

Distribution

+

-

Position values represent number of bases from the 5 prime end of the sequence provided to MEME and the length of that sequence. It is these figures expressed as proportions that are used for statistical analysis.

Probability value of comparison of these values to a linear distribution, p = 0.00051

The motif is found 145 times on the + stand and 121 times on the - strand.

Probability value that it is evenly distributed between the two strands, p = 0.079

Move the mouse pointer over the gene ids to highlight the corresponding marker
